# A young traveller presenting with typhoid fever after oral vaccination: a case report

**DOI:** 10.1186/1752-1947-7-237

**Published:** 2013-10-07

**Authors:** Martin Grimm, Christoph Lübbert, Joachim Mössner, Sebastian Weis

**Affiliations:** 1 Department of Internal Medicine, Neurology and Dermatology, Medical Intensive Care Unit, University Hospital Leipzig, Liebigstrasse 20, 04103 Leipzig, Germany; 2 Department of Internal Medicine, Neurology and Dermatology, Clinic of Gastroenterology and Rheumatology, University Hospital Leipzig, Liebigstrasse 20, 04103 Leipzig, Germany

## Abstract

**Introduction:**

Typhoid fever is one of the most common vaccine-preventable diseases in travellers returning from tropical destinations. However, immunity and the immune response to infection are barely understood.

**Case presentation:**

We report a case of tyhoid fever in a 29-year-old Caucasian, previously healthy woman who did not develop protective immunity or seroconversion of H or O antibodies neither after vaccination with the oral Ty21 vaccine, nor after infection with *Salmonella typhi*.

**Conclusions:**

This case highlights the insufficiencies of the current vaccination and the lack of a reliable, rapid serologic diagnostic tool for typhoid fever. With this case report, we aim to sensitize the reader that typhoid fever has to be taken into account as a differential diagnosis in patients even after vaccination and with negative serological test results.

## Introduction

Enteric fever caused by *Salmonella enterica serovar typhi* is the most common bacteraemic and vaccine-preventable disease in travellers returning from the tropics [1,2]. It is a rare diagnosis in the western world. In Germany for example, there were less than 80 cases annually during the last few years [3]. Nevertheless, typhoid fever has to be considered as a differential diagnosis especially in patients with a travel history to high-endemic areas such as India. Prevention and diagnosis of typhoid fever are hampered as immunity and the immune response to infection are barely understood.

## Case presentation

A 29-year-old female German law student was referred to our outpatient department (OPD) with a two-week history of severe frontal headache and high-grade fever reaching 41°C (106°F). Upon her first presentation, diarrhea, bloody discharge or abdominal cramps were denied. She did not report any weight changes or high-risk sexual behaviour. She had no previous history of diseases and was not on any medication. Paracetamol had relieved pain and fever for up to eight hours.

Her travel history was remarkable for a three-month sojourn to Delhi, India, where she had completed an internship in an upper-class neighbourhood and from which she had returned three weeks prior to presentation. During the last months, she had not taken any antibiotics and was not on malaria prophylaxis. Before travelling, she had received all recommended vaccinations including for typhoid fever (Ty21a) and hepatitis A. Her clinical examination was unremarkable. Routine laboratory tests showed a mild thrombocytopenia of 112Gpt/L (150 to 300) whilst leukocyte counts were not elevated. Her rapid malaria test results were negative. Serum antibodies against typhoid fever (O and H antigens, cutoff 1:200), Serum antibodies against typhoid fever (O and H antigens, cutoff 1:200), human immunodeficiency virus (HIV), herpes simplex virus (HSV) 1/2, dengue fever, and hepatitis C could not be detected. Stool microscopy, stool culture and specific antigene assays were unremarkable for pathologic bacteria, viral antigens, worms or protozoa. As she had no fever at the time of presentation, the attending physician did not take blood cultures.

However, she returned to the OPD five days later as her fever and headaches had not ceased. She then also complained of a non-productive cough, bone and muscle pain, abdominal discomfort and constipation. Blood cultures were taken and she was admitted to the gastroenterology ward.

**Figure 1:**
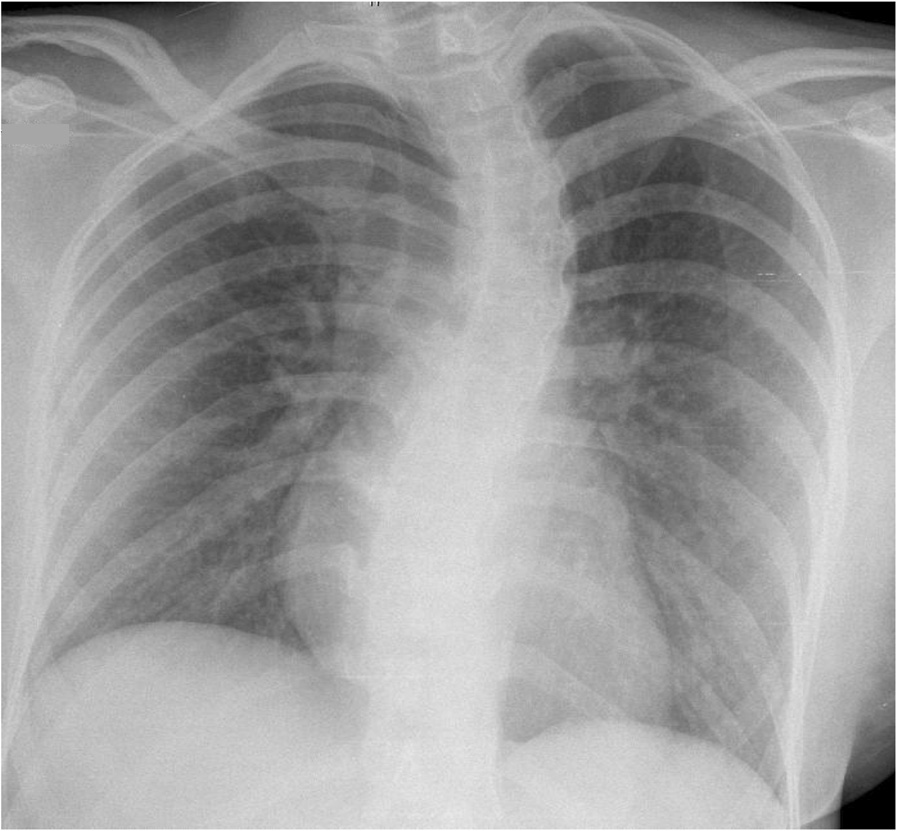
**Chest X-ray taken at the first presentation showing milky opacifications in the basal parts of both lungs as well as a scoliosis.** Courtesy of Prof. Kahn, Department of Imaging, University Hospital Leipzig.

On clinical examination, we saw a deterioration in the young woman’s general and nutritional condition. Her body temperature was 38.3°C (101°F); her heart rate was 90/min with a blood pressure of 115/70mmHg. There was no lymph node swelling. Her sclera were white and without suffusions. Her chest examination result was unremarkable. Her abdomen was slightly tense with reduced bowel sounds. There was no liver or spleen enlargement. Her laboratory test results were remarkable for elevated C-reactive protein (CRP) 142mg/L (<5); aspartate aminotransferase (ASAT) 8μkat/L; alanine aminotransferase (ALAT) 5μkat/L (both <0.6); lactate dehydrogenase (LDH) 17μkat/L (2.2 to 3.5); a decreased thrombocyte count of 109/μL and a cholinesterase activity of 58μkat/L (71 to 181). There was no eosinophilia, and microscopic differentiation showed normal leucocyte values (4.8Gpt/L (4.0 to 9.0), neutrophils 66.6%, lymphocytes 27.5%, monocytes 5.5%, eosinophils 0.0%, basophils 0.4%). Her chest X-ray revealed milky opacities of the basal parts of the lung as well as a prominent scoliosis (Figure 1). A urinary tract infection was ruled out. Stool microscopy and culture were again negative. A calculated antibiotic regimen with intravenous ciprofloxacin 500mg twice daily was initiated and switched to an oral formula after three days. Her antibody test results (including the Widal test) were again negative.

Five days later, three out of four blood cultures returned positive for *Salmonella enterica serovar typhi*. The pathogen was found to be sensible to all tested antibiotics including beta-lactams, fluoroquinolones and cotrimoxazole. Chloramphenicol sensibility was not tested. On the same day, the fever decreased and the headaches resolved. Repeated stool controls were negative for *Salmonella spp*. The patient was discharged after seven days in an improved condition. In order to control *Salmonella* carriage further, stool controls were performed by German health authorities. Only one out of six stool samples turned out to be positive for *Salmonella typhi*. Up to now, the patient remains healthy and has not experienced any typhoid relapse.

## Discussion

Despite being rarely seen in western world hospitals, infection with *S. typhi* remains a global health issue and an important differential diagnosis in patients the return from tropical destinations. The World Health Organization (WHO) estimated 22 million cases and 200,000 deaths per year worldwide [4].

Typhoid fever can be prevented by vaccination but protection rates of the available vaccines are far from satisfying. Although well-working vaccines have been developed for other infectious diseases such as smallpox or polio without understanding the underlying immunology, this has been proven to be difficult for typhoid fever. There are three vaccines licensed of which two are commercially available. Currently, a life-attenuated vaccine strain (Ty21a) that lacks the virulent Vi antigen and a parenteral Vi polysaccharide vaccine are used [5,6]. A recent Cochrane meta-analysis identified 17 randomized trials about typhoid vaccines and showed both vaccines to be equally effective. Three doses of the oral Ty21a vaccine provided a protection rate of 34 to 58%. While the parenteral vaccine showed a cumulative efficacy of 30 to 70% for two years [7]. The Vi vaccine is recommended by the WHO, although controversies on its safety and efficacy in younger children exist [8,9]. Of note, these efficacy rates cannot be directly used for travellers as the data was obtained from local populations. Only an early trial with 20 participants calculated a 95% protection rate in travellers [10].

In endemic countries, the diagnosis of typhoid fever is often based upon the clinical presentation [5]. The diagnostic gold standard remains the bacterial culture especially from the bone marrow area [4]. The classical serologic test - still used in many parts of the world - was first described in 1896 by Felix Widal (the Widal test). This agglutination assay uses the H (flagellar), O (somatic) - and in newer versions also the Vi capsular antigens. Its easy and cheap usage is counterbalanced by unsatisfying sensitivity and specificity [11,12]. In general, a four-time rise within seven to ten days in the agglutinin titre is considered to be a positive test result [13]. Interestingly, *S. typhi* and the attenuated Ty21 vaccine strain both express H and O antigens [5]. In contrast, vaccination with the attenuated vaccine strain resulted in a positive agglutinin test in less than 60% of the participants, only [13]. Moreover, other infectious diseases such as dengue fever or malaria can cause false positive results and positive test results might reflect previous infection. In a review about diagnostic tests for typhoid fever, Olopoenia *et al.* advised that the Widal test in endemic areas should only be considered positive when a fourfold increase in titres within two to three weeks is observed and argues against the usefulness of a single test [14].

As stated by the WHO 40 years ago, '… host defence mechanisms in human typhoid infection are poorly understood and the nature of protective immunity is largely unknown…’ [15]. To date, no correlation of antibody titres to protection against infection or disease could be established and the role of antibodies is unclear [16]. Sarasombath *et al.* investigated the antibody response after typhoid infection and measured anti-O and anti-H agglutinins using the Widal test. All patients developed anti-O agglutinin between week eight and week ten of the disease. But there were three patients with no seroconversion by week four of infection. Moreover anti-H agglutinin was found in all but two patients at week four of the disease [17]. The authors proposed that the presence of O- and H-antigens do not provide full protection and postulated a crucial role for a cellular-mediated immune response [17]. In another early work by Dham *et al.* immune responses after parenteral vaccination and infection were investigated. Although the authors did not especially report the number of patients without seroconversion, apparently, there was at least one patient without the expected rise in H- and O-antibody titres [18]. Thus, some patients, including our case, fail to develop antibody titres. Recently, Lindow *et al.* reported a complex action of typhoid-specific antibodies after vaccination using a new single-dose oral vaccine (M01ZH09) that is under investigation but not yet licensed [16]. In their model, antibodies generated against the vaccine strain resulted in increased uptake and killing of *S. typhi* in macrophage cells and increased complement-mediated killing of the bacteria [16].

Interestingly, *S. typhi* and the attenuated Ty21 vaccine strain both express H- and O-antigens [5]. In the case presented, our patient did not develop an antibody response against these antigens although being exposed twice - once upon vaccination and once upon infection.

Moreover, other infectious diseases such as dengue fever or malaria can cause false positive results and positive test results might reflect previous infection. In their review about diagnostic tests for typhoid fever, Olopoenia *et al.* advised against the routine usage of the Widal test [14].

## Conclusions

We report a case of a young otherwise healthy traveller who had *Salmonella* bacteraemia and clinical signs of systemic infection. She presented with typical clinical signs of typhoid fever such as high fever, constipation and a dry cough. Initial laboratory diagnostics showed eosinophilia and a relatively low leucocyte count (no neutropenia) as a potential diagnostic marker for typhoid fever. Although being vaccinated, she did not develop a protective immunity nor antibody titres against H- and O- antigens after infection. After all, our case underscores the need for a better understanding of the immune responses that occur in typhoid fever. Recent advances and the development of a new animal model of typhoid fever are promising. Using a chimeric *rag-2* deficient mouse with humanized hematopoietic stem and progenitor cells [19] may help to shed light on the so far insufficiently understood immune response and development of immunity to typhoid fever and finally lead to more reliable tests and better vaccines.

## Consent

Written informed consent was obtained from the patient for publication of this case report and any accompanying images. A copy of the written consent is available for review by the Editor-in-Chief of this journal.

## Competing interests

The authors declare that they have no competing interests.

## Authors’ contributions

MG and SW collected the data and drafted the manuscript. CL contributed to the clinical and scientific content of the work. JM critically revised the manuscript. All authors read and approved the final manuscript.
